# Inhibitory non-invasive brain stimulation to homologous language regions as an adjunct to speech and language therapy in post-stroke aphasia: a meta-analysis

**DOI:** 10.3389/fnhum.2015.00236

**Published:** 2015-04-28

**Authors:** Begonya Otal, Manuel C. Olma, Agnes Flöel, Ian Wellwood

**Affiliations:** ^1^Center for Stroke Research Berlin, Charité University MedicineBerlin, Germany; ^2^Department of Neurology, NeuroCure Clinical Research Center, Charité University MedicineBerlin, Germany

**Keywords:** non-invasive brain stimulation, rTMS, tDCS, stroke, aphasia, neurorehabilitation, speech and language therapy

## Abstract

Chronic communication impairment is common after stroke, and conventional speech and language therapy (SLT) strategies have limited effectiveness in post-stroke aphasia. Neurorehabilitation with non-invasive brain stimulation techniques (NIBS)—particularly repetitive transcranial magnetic stimulation (rTMS) or transcranial direct current stimulation (tDCS)—may enhance the effects of SLT in selected patients. Applying inhibitory NIBS to specific homologous language regions may induce neural reorganization and reduce interhemispheric competition. This mini review highlights randomized controlled trials (RCTs) and randomized cross-over trials using low-frequency rTMS or cathodal tDCS over the non-lesioned non-language dominant hemisphere and performs an exploratory meta-analysis of those trials considered combinable. Using a random-effects model, a meta-analysis of nine eligible trials involving 215 participants showed a significant mean effect size of 0.51 (95% *CI* = 0.24–0.79) for the main outcome “accuracy of naming” in language assessment. No heterogeneity was observed (*I*^2^ = 0%). More multicenter RCTs with larger populations and homogenous intervention protocols are required to confirm these and the longer-term effects.

## Introduction

Post-stroke aphasia accounts for around 85% of all cases of aphasia, is present in 21–38% of post-stroke patients (Laska et al., 2001; Berthier, 2005), and poses a major challenge in neurorehabilitation. While spontaneous post-stroke aphasia recovery occurs, this largely takes place in the first 2–3 months after a stroke with a slower rate and longer progress time compared with spontaneous motor recovery (Sarno and Levita, 1981; Wade et al., 1986). Further, 12% of post-stroke survivors are left with some degree of chronic communication deficit even after vigorous treatment (Wade et al., 1986; Lazar et al., 2010). Patients with post-stroke aphasia experience longer length of stays, greater morbidity, and greater mortality than those without aphasia and therefore incur greater costs (Ellis et al., 2012). Additionally, people with aphasia tend to participate in fewer activities and report worse quality of life after stroke than those without aphasia (Hilari, 2011).

The aphasic population is heterogeneous, with individual profiles of language impairment varying in terms of severity and degree of involvement across the modalities of language processing, including the expression and comprehension of speech, reading, writing and gesture (Parr et al., 1997; Code and Herrmann, 2003). Speech and language therapy (SLT) is the most commonly employed treatment in aphasia. Generally, SLT is tailored to meet the individual needs of patients. Nevertheless, its therapeutic effects are quite variable and usually modest (Brady et al., 2012).

Repetitive transcranial magnetic stimulation (rTMS) and transcranial direct current stimulation (tDCS) may be viable approaches to augment the clinical efficacy of conventional SLT strategies. Two separate meta-analyses of published randomized controlled trials (RCTs) in post-stroke aphasia rehabilitation have recently evaluated the clinical efficacy of tDCS (Elsner et al., 2013) and rTMS (Ren et al., 2014). Their results were reported by combinable outcome measures and treatment protocols with subgroup analyses in terms of stimulation type, site of stimulation and duration of stroke. However, both reviews concluded that the evidence base for the effectiveness of these non-invasive brain stimulation (NIBS) techniques remains limited. Existing studies show a remarkable heterogeneity in treatment protocols (e.g., target brain region, stimulation type, frequency, etc.) and variety of aphasic assessment scales. Further, there are no double-blinded RCTs with large populations that demonstrate benefit of rTMS or tDCS as an adjunct to SLT in the long-term recovery of post-stroke aphasia.

The goal of this mini review is to identify and summarize RCTs and randomized controlled cross-over trials assessing the clinical efficacy of NIBS techniques in their inhibitory form (i.e., low-frequency rTMS or cathodal tDCS) over the unaffected non-language dominant hemisphere as an adjunct to SLT for post-stroke aphasia rehabilitation, and where outcome measures are considered comparable, to combine these in an exploratory meta-analysis. This study allows us to specifically examine the neuroplastic process underlying aphasia recovery in adults considering the concept of reducing interhemispheric competition.

## Studies of NIBS in post-stroke aphasia neurorehabilitation

The language network includes (i) Broca's [i.e., pars opercularis (POp)—corresponding to Brodmann Area 44 or BA44—and, pars triangularis (PTr, BA45) on the inferior frontal gyrus (IFG)] and Wernicke's (on the superior temporal gyrus) areas in the left hemisphere (dominant hemisphere); (ii) homologous areas in the right side of the brain (non-dominant hemisphere); (iii) the prefrontal and premotor areas in the frontal regions; and, (iv) the lower part of the parietal region (Vigneau et al., 2006; Frey et al., 2008). Recent neuroimaging studies on post-stroke aphasia revealed maladaptive cortical changes in both hemispheres, yet their functional contribution in language recovery remains elusive (Khedr et al., 2014). Language recovery after a stroke depends significantly on the degree of neuroplastic change, which is usually associated with reorganization and reconnection of the lesioned and perilesional dominant hemisphere regions, acquisition or unmasking of the homologous language area in the non-dominant hemisphere, or activation of the non-dominant cortical region (Hamilton et al., 2011). Heiss and Thiel (2006) postulated that the homologous area in the non-dominant hemisphere of aphasia patients may take over the role of the affected language area of the left hemisphere after a stroke, particularly among patients with extensive left hemisphere injury. However, recent findings have indicated that an upsurge in right hemisphere activity following a stroke may hinder rather than aid recovery (Turkeltaub et al., 2011). Interhemispheric inhibitory connections that normally modulate and effectively suppress right hemispheric activity are disturbed due to damage in the left hemisphere, enabling areas in the contralesional right hemisphere to become increasingly involved via disinhibition. This may exert an inhibitory influence on perilesional areas, negatively affecting spontaneous neuroplasticity and interfering with the ability of perilesional areas to contribute to language recovery. As Shah et al. (2013) further highlighted, this interhemsipheric inhibition model provided the rationale in which suppression of right hemispheric activity or stimulation of the left hemispheric peri-stroke areas with NIBS has been employed in order to enhance language performance in patients with aphasia.

rTMS focal magnetic pulses penetrate the skull to induce weak electrical currents that directly depolarize or hyperpolarize neuronal membranes. rTMS induced currents are sufficient to generate or inhibit action potentials (Pascual-Leone et al., 1998; Fitzgerald et al., 2006). An increasing number of studies have demonstrated that inhibitory low-frequency rTMS (≤1 Hz) over the unaffected hemisphere can be useful in aphasic patients. Ren et al. (2014) identified seven RCTs involving 160 stroke patients for a meta-analysis investigating the effect of low-frequency rTMS mainly targeting the triangular part of the right IFG (Weiduschat et al., 2011; Waldowski et al., 2012; Barwood et al., 2013; Hartmann et al., 2013; Heiss et al., 2013; Seniów et al., 2013; Thiel et al., 2013). These studies tested in randomized sham-controlled studies the concept that downregulating specific intact contralesional cortical areas may help engage more efficient language processes by diminishing the impact of trascallosal imbalance in post-stroke aphasic patients. The underlying mechanisms involved in the application of low-frequency rTMS to selected homologous language regions may include neural reorganization resulting in a prospective reduction in interhemsipheric inhibition (Heiss and Thiel, 2006; Thiel et al., 2006) and an improvement in language recovery. Excitatory or facilatory high-frequency rTMS (>1 Hz) over the damaged hemisphere has also shown improvements in post-stroke aphasia (Szaflarski et al., 2011; Dammekens et al., 2014). Further, Khedr et al. (2014) hypothesized that simultaneous application of low-frequency rTMS over the non-dominant speech area and high-frequency rTMS over the dominant speech area would have a beneficial effect on improving speech performance, particularly if applied early after stroke in combination with language training at the time when neural plasticity might be maximal. The authors emphasized that more neuroimaging studies would be helpful to study bi-hemispheric changes in language recovery.

Like rTMS, tDCS can alter cortical excitability in predictable ways. However, tDCS is characterized as neuromodulatory rather than neurostimulatory, since the currents delivered during tDCS are not sufficient to directly generate or inhibit action potentials. tDCS currents modulate neural resting membrane potentials, in which anodal tDCS (a-tDCS) increases cortical excitability and cathodal (c-tDCS) decreases cortical excitability (Nitsche and Paulus, 2000). tDCS can easily be administered during behavioral treatment, and is less expensive and likely to be better accepted by patients than rTMS (Flöel et al., 2011; Flöel, 2014). Implications for clinical practice should be ascertained in larger multicenter trials. Many studies employing tDCS as a therapy for aphasia have adopted approaches that are broadly consistent with an interhemispheric inhibition model of aphasia recovery. That is, a-tDCS investigations are mainly centered on left hemisphere language areas in order to increase the excitability in the perilesional and residual fronto-temporal areas (Baker et al., 2010; Fiori et al., 2011; Fridriksson et al., 2011; Marangolo et al., 2013), whereas c-tDCS is generally applied to the right homotopic areas to inhibit over activation (due to transcollasal disinhibition) in the contralesional right homologs. In a recent Cochrane meta-analysis, Elsner et al. (2013) evaluated five tDCS interventional trials with sham-controls involving 54 post-stroke aphasic patients. Although these studies using tDCS in combination with SLT favored the intervention in each of these five trials (Monti et al., 2008; Flöel et al., 2011; Kang et al., 2011; Marangolo et al., 2011; You et al., 2011), confidence interval width did not allow the results to be generalized. Elsner et al. (2013) did however state that when considering only c-tDCS over the non-lesioned hemisphere vs. sham-tDCS, the effect on naming accuracy rises and the probability of error declines.

As far as we know, no other meta-analysis of RCTs or randomized controlled cross-over trials has been conducted to examine the effects of two inhibitory NIBS techniques, here low-frequency rTMS and c-tDCS, using treatment protocols and outcome measures considered combinable.

## Methods

Electronic searches were performed in PubMed, Embase and *ClinicalTrials.gov* databases and limited to studies written in English and published from March 2012, the date of search finalization of the previous meta-analysis (Elsner et al., 2013), up until end September 2014. The search terms were (“repetitive transcranial magnetic stimulation” OR “rTMS” OR “transcranial direct current stimulation” OR “tDCS”) AND (“aphasia” OR “language disorder” OR “anomia”) AND (“stroke” OR “post-stroke”). We also considered previous results from the recent meta-analysis of rTMS (Ren et al., 2014) and tDCS (Elsner et al., 2013). Studies were included if they met the following inclusion criteria:

1. Design: double-blinded RCTs or randomized controlled cross-over trials with at least four participants.
2. Participants: adult patients of either gender diagnosed with ischemic stroke-induced aphasia (no lesion location or chronicity limits).
3. Intervention: inhibitory NIBS technique (i.e., low-frequency rTMS or c-tDCS) over the unaffected non-language dominant hemisphere as an adjunct to SLT.
4. Control group: sham NIBS (i.e., sham rTMS or sham tDCS, respectively) with SLT.
5. Outcome measures were reported with continuous scales that evaluated the accuracy of naming (as degree of language impairment) immediately after treatment.

One review author (BO) read the titles and abstracts of the records identified from the electronic searches and eliminated obviously irrelevant studies. Two independent authors (MO and BO) examined whether the potentially relevant publications fitted our inclusion criteria and assessed them for methodological quality and risk of bias using the 11 item PEDro^1^ scale (Maher et al., 2003). The PEDro scale rates the methodological quality of randomized trials out of 10. Item 1 is related to the external validity and therefore not included in the total PEDro score (PEDro). Eligible studies scoring ≥6 out of 10 were considered to be high quality and qualified for quantitative synthesis.

Number of participants, means and standard deviations of the outcome measures that evaluated the accuracy of naming were extracted. Trials using similar methods of measurement immediately after treatment were considered for pooling. Then, the data were entered into the Review Manager software (RevMan 5.3)^2^ and pooling was carried out for statistical analyses. Since different methods of measurement were used across studies, the effect sizes (ES) were reported as standardized mean differences (SMD) and 95% confidence intervals (CIs), and a random effects model was used because it provides a more conservative ES estimate. The heterogeneity across each ES was evaluated with the *I*^2^ statistic, and this review considered 25% low, 50% moderate, and 75% high (Higgins et al., 2003).

## Results

### Results of literature search and main characteristics

We identified 67 unique records from the database searches (see Supplementary Figure). After further assessments, we excluded 62 studies that did not meet the inclusion criteria, or, were ongoing clinical trials or with unpublished results. Five RCTs published from March 2012 to September 2014 were eligible (Waldowski et al., 2012; Heiss et al., 2013; Seniów et al, 2013; Thiel et al., 2013; Tsai et al., 2014). From the previous meta-analysis by Elsner et al. (2013) of tDCS and Ren et al. (2014) of rTMS, we included four additional interventional trials (Flöel et al., 2011; Kang et al., 2011; Weiduschat et al., 2011; You et al., 2011) that met our criteria. The assessment of risk of bias showed that all studies had PEDro scores of ≥6, indicating consistent methodological quality and a low risk of most biases (see Supplementary Table). Overall, nine high quality interventional trials involving 215 participants were retained for quantitative synthesis: six RCTs of low-frequency rTMS [five previously identified by Ren et al. (2014) and one recently published by Tsai et al. (2014)] and three interventional trials of c-tDCS identified by Elsner et al. (2013). Table 1 summarizes the main characteristics of all the inhibitory NIBS included studies.

**Table 1:** **Characteristics of inhibitory NIBS included studies**.

**Authors and years**	**Subjts # (Exp/Ctr)**	**Mean Age, Y (Exp/Ctr)**	**Stroke stage^c^**	**First lang**.	**Aphasia type**	**Main outcome**	**NIBS protocol + SLT *(# days/sessions)***	**Position**
**^a^LOW-FREQUENCY rTMS vs. SHAM rTMS**								
Heiss et al., 2013	29 (15/14)	(68.5/69)	Subacute	German	Varying type	AAT naming subtest	1 Hz rTMS, 90% rMT, (20 min). Immediately after, +45 min SLT (*10 days/sessions*)	Right IFG (pars triangularis, PTr)
Seniów et al., 2013	38 (19/19)	(61.8/59.7)	Subacute	Polish	Varying type	BDAE naming subtest	1 Hz rTMS, 90% rMT, (30 min). Immediately after, +45 min SLT (*15 days/sessions*)	Right IFG (pars triangularis, PTr)
Thiel et al., 2013	24 (13/11)	(69.8/71.2)	Subacute	Geman	Varying type	AAT naming subtest	1 Hz rTMS, 90% rMT, (20 min). Immediately after, +45 min SLT (*10 days/sessions*)	Right IFG (pars triangularis, PTr)
Tsai et al., 2014	56 (33/23)	(62.3/62.8)	Chronic	Chinese	non-fluent	PNT accuracy of naming	1 Hz rTMS, 90% rMT, (10 min). After max. 30 min, +60 min SLT (*10 days/sessions*)	Right IFG (pars triangularis, PTr)
Waldowski et al., 2012	26 (13/13)	(62.3/60.1)	Subacute	Polish	Varying type	CPNT accuracy of naming	1 Hz rTMS, 90% rMT, (30 min). Immediately after, +45 min SLT (*15 days/sessions*)	Right IFG (pars triangularis, PTr and pars opercularis, POp)
Weiduschat et al., 2011	10 (6/4)	(66.6/63.7)	Subacute	German	varying type	AAT naming subtest	1 Hz rTMS, 90% rMT, (20 min). Immediately after, +45 min SLT (*10 days/sessions*)	Right IFG (pars triangularis, PTr)
**^b^CATHODAL tDCS vs. SHAM tDCS**								
Flöel et al., 2011	8 (8/8)	(52.3/52.3)	Chronic	German	Varying type	Accuracy of naming sp. task	1 mA c-tDCS (2 × 20 min) within 120 min SLT (*3 days/sessions*)	Right temporo-parietal cortex
Kang et al., 2011	10 (5/5)	(62/61.8)	Chronic	Korean	Varying type	BNT accuracy of naming	2 mA c-tDCS (20 min) within 30 min SLT (*5 days/sessions*)	Right IFG (Broca's homolog)
You et al., 2011	14 (7/7)	(68.1/63.4)	Subacute	Korean	Global	K-WAB aphasia quotient	2 mA c-tDCS (30 min) within SLT (*10 days/sessions*)	Right superior temporal gyrus (Wernicke's homolog)

*Low-frequency rTMS (≤1 Hz) and cathodal tDCS (c-tDCS) over the non-lesioned non-language dominant hemisphere in combination with SLT*.

*AAT, Aachen Aphasia Test; BDAE, Boston Diagnostic Aphasia Examination; BNT, Korean Boston Naming Test; CPNT, Computerized Picture Naming Test; IFG, Inferior Frontal Gyrus; PNT, Picture Naming Test; K-WAB, Korean-Western Aphasia Battery*.

a *Low-frequency rTMS trials were evaluated in Ren et al. (2014) with the exception of Tsai et al., 2014*.

b *Cathodal tDCS trials were evaluated in Elsner et al. (2013)*.

c *Stroke stage: subacute (7 d to 3 mo); chronic (>3 mo)*.

### Included trials of low-frequency rTMS

We included six RCTs involving 183 participants investigating the effect of low-frequency rTMS over the non-lesioned hemisphere (experimental group) vs. sham rTMS (control group) in combination with SLT. Three of the included studies were conducted with German-speakers and used the Aachen Aphasia Test (AAT) as an outcome measure (Weiduschat et al., 2011; Heiss et al., 2013; Thiel et al., 2013), two with Polish-speakers using an adapted version of the Boston Diagnostic Aphasia Examination (BDAE) (Seniów et al., 2013) and the Computerized Picture Naming Test (CPNT) (Waldowski et al., 2012). Finally, one was conducted in Chinese using the Picture Naming Test (PNT) as a main outcome measure (Tsai et al., 2014). All studies measured the degree of “accuracy of naming” performance.

### Included trials of cathodal tDCS

We included three interventional trials involving 32 participants examining the effect of c-tDCS over the non-lesioned hemisphere (experimental group) vs. sham tDCS (control group) in combination with SLT. Two of these three studies were randomized cross-over trials (Flöel et al., 2011; Kang et al., 2011), one was a RCT (You et al., 2011). Two of the included studies were conducted in the Republic of Korea using a standardized, validated Korean version of the Boston Naming Test (BNT) (Kang et al., 2011) and the Western Aphasia Battery (You et al., 2011) as outcome measures. One further study was conducted with German speakers and the main outcome parameter was the naming ability for trained objects (Flöel et al., 2011). All studies measured the degree of “accuracy of naming” performance.

### Results of the meta-analysis

**Figure 1:**
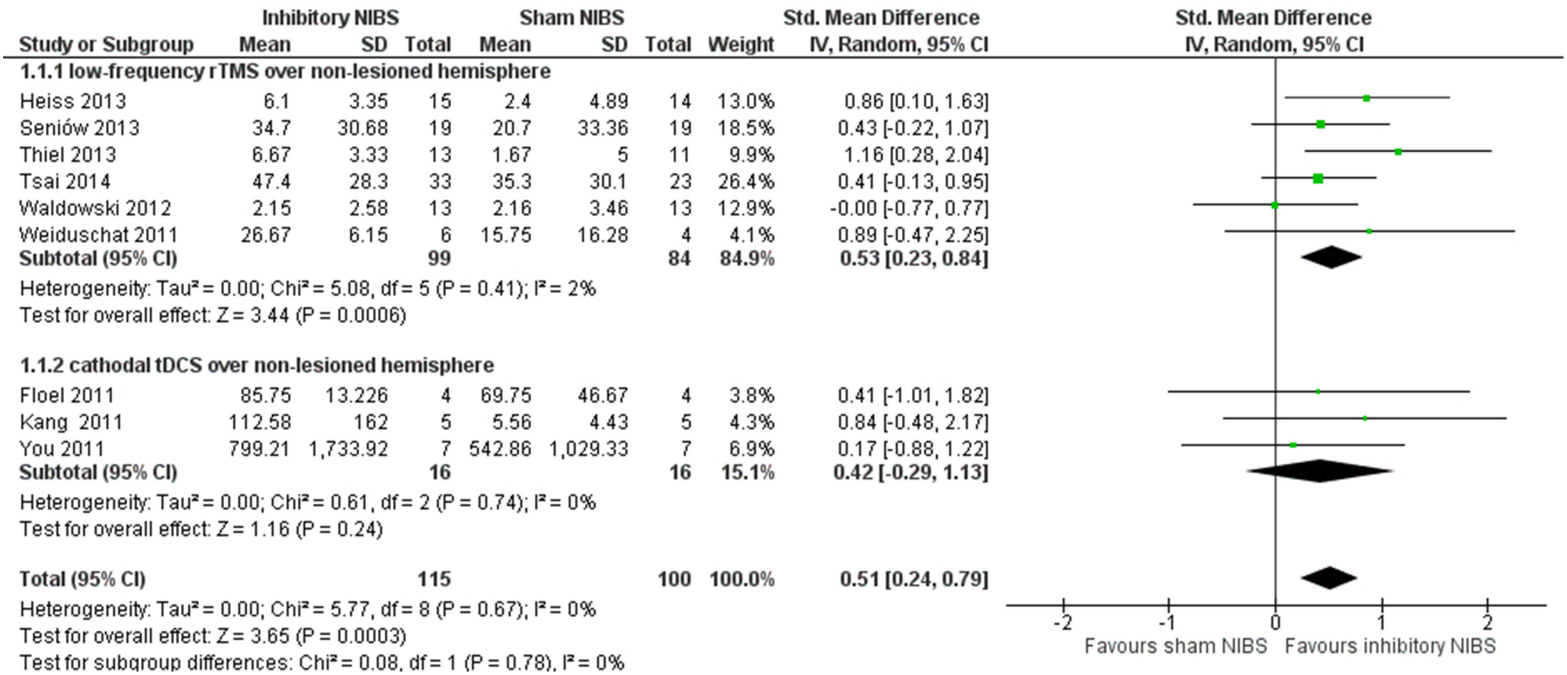
**Meta-analysis by inhibitory non-invasive brain stimulation (NIBS) (low-frequency rTMS and cathodal tDCS) vs. sham NIBS over the non-lesioned non-language dominant hemisphere**. Forest plot of SMD and 95% CI for the outcome of accuracy of naming (relative change in per cent) in post-stroke aphasic patients until end of intervention phase, inhibitory NIBS vs. sham NIBS. All included trials utilized inhibitory NIBS and sham NIBS in combination with SLT.

Figure 1 illustrates a forest plot of the SMD for the main outcome “accuracy of naming” of the corresponding language assessment scales (Table 1). The overall “accuracy of naming” score was significantly improved in patients receiving inhibitory NIBS over the non-lesioned hemisphere as an adjunct to SLT compared to sham-NIBS controls (with SLT), with a significant mean effect size of 0.51 (95% *CI* = 0.24–0.79, *P* = 0.0003). Between study heterogeneity was negligible (*I*^2^ = 0%).

## Discussion

Our work complements recent systematic reviews of rTMS (Ren et al., 2014) and tDCS (Elsner et al., 2013) and enables for the first time a preliminary comparison of the effect size of two inhibitory NIBS techniques in clinical studies using combinable protocols and outcome measures.

Our results are coherent with the concept that inhibitory NIBS to selected right hemispheric homologous language regions induce neural reorganization and reduce interhemispheric competition. A higher significant SMD of 0.53 was found if we consider only the subgroup of trials with low-frequency rTMS (183 participants) targeting the right IFG (mainly PTr). There is some indication that an inhibitory NIBS effect might be more relevant on the right homologs of Broca's area, right PTr but not POp (Naeser et al., 2011), which most rTMS RCTs target exclusively, with the exception of Waldowski et al. (2012) that also included POp.

The lower SMD with a wide confidence interval in c-tDCS trials indicates no statistically significance to generalize its effect. However, the mean effect size in this subgroup is greater than that seen when stimulating with a-tDCS over the lesioned language-dominant hemisphere (Elsner et al., 2013), where no effect was found. It should be noted that the number of participants in c-tDCS trials is still very limited (only 32 participants) and that the location site varied considerably among the different studies. Only Kang et al. (2011) targeted the right IFG (right Broca's homolog) similar to the included rTMS trials. To further analyze this issue is beyond of the scope of this review.

Our results indicate low levels of heterogeneity between studies in the meta-analysis with low *I*^2^%. Nevertheless, some may consider there to be considerable variability of types of aphasia, study design, stimulation protocol and site. Where this is the case it can be difficult to assess combinability of studies. We acknowledge this difficulty and recommend cautious interpretation of our exploratory analysis. Not all aphasic patients may benefit from inhibitory NIBS after stroke, but no adverse events were reported in the included trials. Our study has several limitations: (1) we did not include any unpublished or non-English language studies; (2) too few inhibitory NIBS trials were included to generalize the results; (3) publication and selection bias might have affected our results.

This exploratory meta-analysis suggests potential benefits of low-frequency rTMS and c-tDCS over right hemispheric homologous language regions in post-stroke aphasia, though the effects of c-tDCS still need to be confirmed in RCTs with larger cohorts of patients and more homogenous protocols. Further RCTs of c-tDCS modulating the right Broca's homolog (especially the right PTr) in the subacute phase may be relevant to allow more realistic comparisons vs. low-frequency rTMS. Additionally, RCTs directly comparing low-frequency rTMS vs. c-tDCS may provide insight in assessing which inhibitory technique is most clinically effective and best tolerated. More well-designed longitudinal studies and standardized region location methods are necessary to determine the effect duration and long-term impact in language recovery of both techniques.

## Conclusion

There is a lack of information comparing the clinical efficacy of trials of rTMS and tDCS as an adjunct to conventional SLT utilizing combinable protocols and outcome measures in post-stroke aphasia. By using stringent inclusion criteria, this exploratory meta-analysis combines existing randomized trials of these two inhibitory NIBS techniques in combination with SLT. Our results reflect that low-frequency rTMS and c-tDCS over the unaffected non-language dominant hemisphere may be a promising approach compatible with the concept of interhemispheric inhibition. Further multicenter RCTs with larger populations and homogenous intervention protocols are required to confirm these and the longer-term effects.

### Conflict of interest statement

AF was the principle investigator on one of the trials reviewed. During the time of the trial, AF was supported by German Science Foundation (Fl-379-4/2 379-8/1) and the Federal Ministry for Education and Science (FKZ 0315673A). The authors declare that the research was conducted in the absence of any commercial or financial relationships that could be construed as a potential conflict of interest.

## Footnotes

^1^PEDro. Physiotherapy Evidence Database [http://www.pedro.org.au/].

^2^RevMan. (2011). *Review Manager*5.3. Copenhagen: The Nordic Cochrane Centre, The Cochrane Collaboration.

## References

[B1] Baker J. M. Rorden C. Fridriksson J. (2010). Using transcranial direct-current stimulation to treat stroke patients with aphasia. Stroke 41, 1229–1236. 10.1161/STROKEAHA.109.576785 20395612 PMC2876210

[B2] Barwood C. H. Murdoch B. E. Riek S. O'Sullivan J. D. Wong A. Lloyd D.. (2013). Long term language recovery subsequent to low frequency rTMS in chronic non-fluent aphasia. Neurorehabilitation 32, 915–928. 10.3233/NRE-130915 23867417

[B3] Berthier M. L. (2005). Postroke aphasia—epidemiology, pathophysiology and treatment. Drugs Aging 22, 163–182. 10.2165/00002512-200522020-00006 15733022

[B4] Brady M. C. Kelly H. Godwin J. Enderby P. (2012). Speech and language therapy for aphasia following stroke. Cochrane Database Syst. Rev. 5:CD000425. 10.1002/14651858.CD000425 20464716

[B5] Code C. Herrmann M. (2003). The relevance of emotional and psychosocial factors in aphasia to rehabilitation. Neuropsychol. Rehabil. 13, 109–132. 10.1080/09602010244000291 21854330

[B6] Dammekens E. Vanneste S. Ost J. De Ridder D. (2014). Neural correlates of high frequency repetitive transcranial magnetic stimulation improvement in post-stroke non-fluent aphasia: a case study. Neurocase 20, 1–9. 10.1080/13554794.2012.713493 22963195

[B7] Ellis C. Simpson A. N. Bonilha H. Mauldin P. D. Simpson K. N. (2012). The one-year attributable cost of poststroke aphasia. Stroke 43, 1429–1431. 10.1161/STROKEAHA.111.647339 22343643 PMC4507407

[B8] Elsner B. Kugler J. Pohl M. Mehrholz J. (2013). Transcranial direct current stimulation (tDCS) for improving aphasia in patients after stroke. Cochrane Database Syst. Rev. 6:CD009760. 10.1002/14651858.CD009760 23799617

[B9] Fiori V. Coccia M. Marinelli C. V. Vecchi V. Bonifazi S. Ceravolo M. G.. (2011). Transcranial direct current stimulation improves word retrieval in healthy and nonfluent aphasic subjects. J. Cogn. Neurosci. 23, 2309–2323. 10.1162/jocn.2010.21579 20946060

[B10] Fitzgerald P. B. I. Fountain S. Daskalakis Z. J. (2006). A comprehensive review of the effects of rTMS on motor cortical excitability and inhibition. Clin. Neurophysiol. 117, 2584–2596. 10.1016/j.clinph.2006.06.712 16890483

[B11] Flöel A. (2014). tDCS-enhanced motor and cognitive function in neurological diseases. Neuroimage 85 (Pt 3), 934–947. 10.1016/j.neuroimage.2013.05.098 23727025

[B12] Flöel A. Meinzer M. Kirstein R. Nijhof S. Deppe M. Knecht S.. (2011). Short-term anomia training and electrical brain stimulation. Stroke 42, 2065–2067. 10.1161/STROKEAHA.110.609032 21636820

[B13] Frey S. Campbell J. S. Pike G. B. Petrides M. (2008). Dissociating the human language pathways with high angular resolution diffusion fiber tractography. J. Neurosci. 28, 11435–11444. 10.1523/JNEUROSCI.2388-08.2008 18987180 PMC6671318

[B14] Fridriksson J. Richardson J. D. Baker J. M. Rorden C. (2011). Transcranial direct current stimulation improves naming reaction time in fluent aphasia: a double-blind, sham-controlled study. Stroke 42, 819–821. 10.1161/STROKEAHA.110.600288 21233468 PMC8210639

[B15] Hamilton R. H. Chrysikou E. G. Coslett B. (2011). Mechanisms of aphasia recovery after stroke and the role of noninvasive brain stimulation. Brain Lang. 118, 40–50. 10.1016/j.bandl.2011.02.005 21459427 PMC3109088

[B16] Hartmann A. Rubi-Fessen I. Heiss W. D. (2013). rTMS in the treatment of post-stroke aphasia. Neurophsysiol. Clin. 43, 70–71 10.1016/j.neucli.2012.11.012

[B17] Heiss W. D. Hartmann A. Rubi-Fessen I. Anglade C. Kracht L. Kessler J.. (2013). Noninvasive brain stimulation for treatment of right- and left-handed poststroke aphasics. Cerebrovasc. Dis. 36, 363–372. 10.1159/000355499 24217362

[B18] Heiss W. D. Thiel A. (2006). A proposed regional hierarchy in recovery of post-stroke aphasia. Brain Lang. 98, 118–123. 10.1016/j.bandl.2006.02.002 16564566

[B19] Higgins J. Thompson S. Deeks J. Altman D. (2003). Measuring inconsistency in meta-analyses. BMJ 327:557–560. 10.1136/bmj.327.7414.557 12958120 PMC192859

[B20] Hilari K. (2011). The impact of stroke: are people with aphasia different to those without? Disabil. Rehabil. 33, 211–218. 10.3109/09638288.2010.508829 20712416

[B21] Kang E. K. Kim Y. K. Sohn H. M. Cohen L. G. Paik N. J. (2011). Improved picture naming in aphasia patients treated with cathodal tDCS to inhibit the right Broca's homologue area. Restor. Neurol. Neurosci. 29, 141–152. 10.3233/RNN-2011-0587 21586821 PMC4886370

[B22] Khedr E. M. Abo El-Fetoh N. Ali A. M. El-Hammady D. H. Khalifa H. Atta H.. (2014). Dual-hemisphere repetitive transcranial magnetic stimulation for rehabilitation of poststroke aphasia: a randomized, double-blind clinical trial. Neurorehabil. Neural. Repair. 28, 740–750. 10.1177/1545968314521009 24503205

[B23] Laska A. C. Hellblom A. Murray V. Kahan T. von Arbin M. (2001). Aphasia in acute stroke and relation to outcome. J. Intern. Med. 249, 413–422. 10.1046/j.1365-2796.2001.00812.x 11350565

[B24] Lazar R. M. Minzer B. Antoniello D. Festa J. R. Krakauer J. W. Marshall R. S. (2010). Improvement in aphasia scores after stroke is well predicted by initial severity. Stroke 41, 1485–1488. 10.1161/STROKEAHA 20538700 PMC2921806

[B25] Maher C. G. Sherrington C. Herbert R. D. Moseley A. M. Elkins M. (2003). Reliability of the PEDro scale for rating quality of randomized controlled trials. Phys. Ther. 83, 713–772. Available online at: http://ptjournal.apta.org/content/83/8/713.full.pdf+html; http://ptjournal.apta.org/content/83/8/713.long 12882612

[B26] Marangolo P. Fiori V. Calpagnano M. A. Campana S. Razzano C. Caltagirone C.. (2013). tDCS over the left inferior frontal cortex improves speech production in aphasia. Front. Hum. Neurosci. 7:539. 10.3389/fnhum.2013.00539 24046740 PMC3764371

[B27] Marangolo P. Marinelli C. V. Bonifazi S. Fiori V. Ceravolo M. G. Provinciali L.. (2011). Electrical stimulation over the left inferior frontal gyrus (IFG) determines long-term effects in the recovery of speech apraxia in three chronic aphasics. Behav. Brain Res. 225, 498–504. 10.1016/j.bbr.2011.08.008 21856336

[B28] Monti A. Cogiamanian F. Marceglia S. Ferrucci R. Mameli F. Mrakic-Sposta S.. (2008). Improved naming after transcranial direct current stimulation in aphasia. J. Neurol. Neurosurg. Psychiatry 79, 451–453. 10.1136/jnnp.2007.135277 18096677

[B29] Naeser M. A. Martin P. I. Theoret H. Kobayashi M. Fregni F. Nicholas M.. (2011). TMS suppression of right pars triangularis, but not pars opercularis, improves naming in aphasia. Brain Lang. 119, 206–213. 10.1016/j.bandl.2011.07.005 21864891 PMC3195843

[B30] Nitsche A. Paulus W. (2000). Excitability changes induced in the human motor cortex by weak transcranial direct current stimulation. J. Physiol. 527 (Pt 3), 633–699. 10.1111/j.1469-7793.2000.t01-1-00633.x 10990547 PMC2270099

[B31] Parr S. Byng S. Gilpin S. Ireland C. (1997). Talking about Aphasia: Living with Loss of Language after Stroke. Buckingham: OUP.

[B32] Pascual-Leone A. I. Tormos J. M. Keenan J. Tarazona F. Cañete C. Catalá M. D. (1998). Study and modulation of human cortical excitability with transcranial magnetic stimulation. J. Clin. Neurophysiol. 15, 333–343. 9736467 10.1097/00004691-199807000-00005

[B33] Ren C. L. Zhang G. F. Xia N. Jin C. H. Zhang X. H. Hao J. F.. (2014). Effect of low-frequency rTMS on aphasia in stroke patients: a meta-analysis of randomized controlled trials. PLoS ONE 9:e102557. 10.1371/journal.pone.0102557 25036386 PMC4103829

[B34] Sarno M. T. Levita E. (1981). Some observations on the nature of recovery in global aphasia after stroke. Brain Lang. 13, 1–12. 7237109 10.1016/0093-934x(81)90124-3

[B35] Seniów J. Waldowski K. Leøeniak M. Iwañski S. Czepiel W. Członkowska A. (2013). Transcranial magnetic stimulation combined with speech and language training in early aphasia rehabilitation: a randomized double-blind controlled pilot study. Top Stroke Rehabil. 20, 250–261. 10.1310/tsr2003-250 23841973

[B36] Shah P. P. Szaflarski J. P. Allendorfer J. Hamilton R. H. (2013). Induction of neuroplasticity and recovery in post-stroke aphasia by non-invasive brain stimulation. Front. Hum. Neurosci. 7:888. 10.3389/fnhum.2013.00888 24399952 PMC3870921

[B37] Szaflarski J. P. Vannest J. Wu S. W. DiFrancesco M. W. Banks C. Gilbert D. L. (2011). Excitatory repetitive transcranial magnetic stimulation induces improvements in chronic post-stroke aphasia. Med. Sci. Monit. 17, CR132–CR139. 10.12659/MSM.881446 21358599 PMC3057942

[B38] Thiel A. Hartmann A. Rubi-Fessen I. Anglade C. Kracht L. Weiduschat N.. (2013). Effects of noninvasive brain stimulation on language networks and recovery in early poststroke aphasia. Stroke 44, 2240–2246. 10.1161/STROKEAHA.111.000574 23813984

[B39] Thiel A. Schumacher B. Wienhard K. Gairing S. Kracht L. W. Wagner R.. (2006). Direct demonstration of transcallosal disinhibition in language networks. J. Cereb. Blood Flow Metab. 26, 1122–1127. 10.1038/sj.jcbfm.9600350 16757978

[B40] Tsai P. Y. Wang C. P. Ko J. S. Chung Y. M. Chang Y. W. Wang J. X. (2014). The persistent and broadly modulating effect of inhibitory rTMS in nonfluent aphasic patients: a sham-controlled, double-blind study. Neurorehabil. Neural. Repair 28, 779–787. 10.1177/1545968314522710 24526709

[B41] Turkeltaub P. E. Messing S. Norise C. Hamilton R. H. (2011). Are networks for residual language function and recovery consistent across aphasic patients? Neurology 76, 1726–1734. 10.1212/WNL.0b013e31821a44c1 21576689 PMC3100133

[B42] Vigneau M. Beaucousinm V. Hervé P. Y. Duffau H. Crivello F. Houdé O.. (2006). Meta-analyzing left hemisphere language areas: phonology, semantics, and sentence processing. Neuroimage 30, 1414–1432. 10.1016/j.neuroimage.2005.11.002 16413796

[B43] Wade D. T. Hewer R. L. David R. M. Enderby P. M. (1986). Aphasia after stroke: natural history and associated deficits. J. Neurol. Neurosurg. Psychiatry 49, 11–16. 2420939 10.1136/jnnp.49.1.11 PMC1028640

[B44] Waldowski K. Seniów J. Leøeniak M. Iwañski S. Członkowska A. (2012). Effect of low-frequency repetitive transcranial magnetic stimulation on naming abilities in early-stroke aphasic patients: a prospective, randomized, double-blind sham-controlled study. Sci. World J. 2012:518568. Available online at: http://www.hindawi.com/journals/tswj/2012/518568/ 10.1100/2012/518568 23213288 PMC3508571

[B45] Weiduschat N. Thiel A. Rubi-Fessen I. Hartmann A. Kessler J. Merl P.. (2011). Effects of repetititive transcranial magnetic stimulation in aphasic stroke: a randomised controlled pilot study. Stroke 42, 409–415. 10.1161/STROKEAHA.110.597864 21164121

[B46] You D. S. Kim D. Y. Chun M. H. Jung S. E. Park S. J. (2011). Cathodal transcranial direct current stimulation of the right Wernicke's area improves comprehension in subacute stroke patients. Brain Lang. 119, 1–5. 10.1016/j.bandl.2011.05.002 21641021

