# Supplementary material for: Inhibitory non-invasive brain stimulation to homologous language regions as an adjunct to speech and language therapy in post-stroke aphasia: a meta-analysis
Source: Front Hum Neurosci. 2015 Apr 28;9:236. doi: 10.3389/fnhum.2015.00236 (PMC4412051; doi:10.3389/fnhum.2015.00236)
Supplement: Supplementary file 1 [file DataSheet1.PDF]

Supplementary Material

Inhibitory non-invasive brain stimulation to homologous language regions as an adjunct to speech and language therapy in post-stroke aphasia: a meta-analysis

Begonya Otal[1]*, Manuel Olma[1], Agnes Flöel[1,2] and Ian Wellwood[1]

[1]Center for Stroke Research Berlin, Charité University Medicine, Berlin, Germany
[2]Department of Neurology, NeuroCure Clinical Research Center, Charité University Medicine, Berlin, Germany

*** Correspondence:**
Dr. Begonya Otal, Center for Stroke Research Berlin, Charité University Medicine, Berlin, Germany
e-mail: begonya.otal@gmail.com

Keywords: Non-invasive Brain Stimulation, NIBS, rTMS, TMS, tDCS, Stroke, Aphasia, Neurorehabilitation, Speech and language therapy

Supplementary Figure and Table for Results of literature search and main characteristics

Supplementary Figure. Flowchart for the inclusion of inhibitory NIBS studies. Low-frequency rTMS (≤ 1 Hz) and cathodal tDCS (c-tDCS) over the non-lesioned non-language dominant hemisphere in combination with SLT.

Supplementary Table. PEDro Scale scores for the NIBS included studies (n=9).

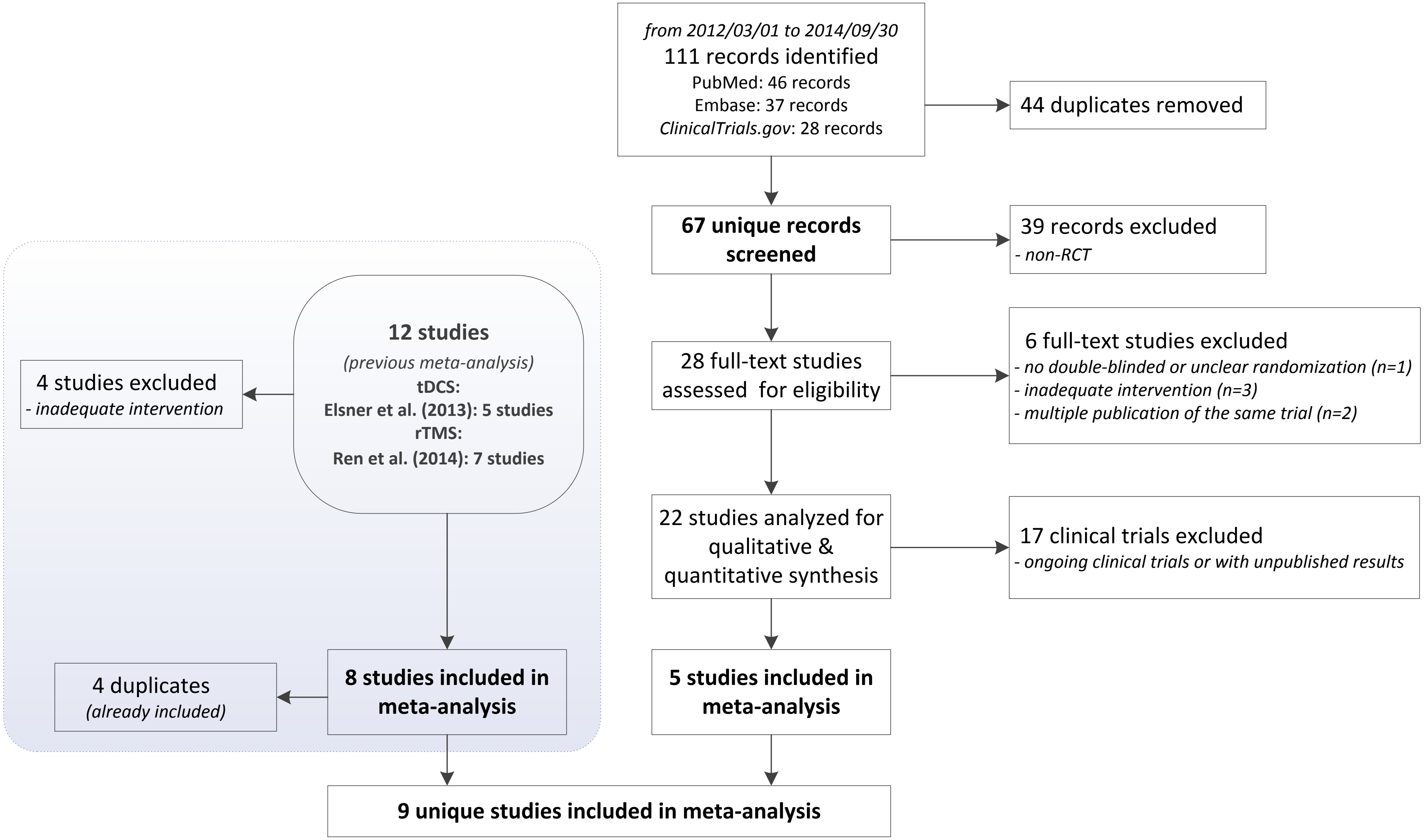

Supplementary Figure. Flowchart for the inclusion of inhibitory NIBS studies. Low-frequency rTMS (≤ 1 Hz) and cathodal tDCS (c-tDCS) over the non-lesioned non-language dominant hemisphere in combination with SLT.

Supplementary Table. PEDro Scale scores for the NIBS included studies (n=9)

Author & year	Random allocation	Concealed allocation	Groups similar at baseline	Participant blinding	Therapists binding	Assessor blinding	< 15% dropouts	Intention-to-treat analysis	Between-group difference reported	Point estimate and variability reported	Total (0 to 10)
**Heiss** 2013	Y	N	Y	Y	N	Y	N	N	Y	Y	6
**Seniów** 2013	Y	Y	Y	Y	N	Y	Y	N	Y	Y	8
**Thiel** 2013	Y	N	Y	Y	N	Y	N	N	Y	Y	6
**Tsai** 2014	Y	Y	Y	Y	N	Y	Y	N	Y	Y	8
**Waldowski** 2012	Y	N	Y	Y	N	Y	Y	Y	Y	Y	8
**Weiduschat** 2011	Y	Y	Y	Y	N	Y	N	N	Y	Y	7
**Floel** 2011	Y	N	Y	Y	N	Y	Y	N	Y	Y	7
**Kang** 2011	Y	N	Y	Y	N	Y	Y	Y	Y	Y	8
**You** 2011	Y	N	Y	Y	N	Y	Y	N	Y	Y	7

N=No, Y=Yes, PEDro= Physiotherapy Evidence Database
